# Balancing Time and Risk: Temporary Arterial Occlusion in Middle Cerebral Artery Aneurysm Surgery

**DOI:** 10.3390/brainsci16050449

**Published:** 2026-04-24

**Authors:** Philipp Geiger, Christian Preuss-Hernandez, Daniel Pinggera, Claudius Thomé, Ondra Petr

**Affiliations:** Department of Neurosurgery, Medical University Innsbruck, 6020 Tyrol, Austria; geiger.philipp@gmx.at (P.G.); medicoblastillo@hotmail.com (C.P.-H.); daniel.pinggera@tirol-kliniken.at (D.P.);

**Keywords:** middle cerebral artery aneurysm, temporary arterial occlusion, surgical clipping, subarachnoid hemorrhage, ischemia, cerebral vasospasm, modified Rankin Scale

## Abstract

**Highlights:**

**What are the main findings?**

- Temporary arterial occlusion (TAO) time was not associated with postoperative ischemic lesions on early imaging after elective microsurgical aneurysm clipping.
- TAO was significantly associated with higher risk for postoperative ischemia in ruptured aneurysm (aSAH) cases, supporting the heightened vulnerability of this subgroup.

**What are the implications of the main findings?**

- TAO clipping should be kept as short as feasible and used in a targeted manner, particularly in aSAH patients.
- The results support future prospective work integrating standardized occlusion protocols and adjunct monitoring (e.g., IONM/perfusion assessment) to refine patient-specific safety limits.

**Abstract:**

**Background:** Temporary arterial occlusion (TAO) is a key adjunct in microsurgical clipping of middle cerebral artery (MCA) aneurysms, but its safe duration and impact on perioperative ischemia—particularly in subarachnoid hemorrhage (SAH)—remain uncertain. **Methods:** A retrospective cohort of 245 patients undergoing microsurgical clipping of MCA aneurysms (154 incidental, 91 SAH) at a tertiary neurovascular center (2010–2020) was analyzed. TAO use, cumulative duration (>5, >8, >10, >15 min), number of applications, and occlusion site were extracted alongside clinical, radiographic, and outcome data. The primary endpoint was perioperative ischemia within 48 h; secondary endpoints included clinically relevant cerebral vasospasm (CVS), intraoperative rupture, and functional outcome (mRS) at discharge and 6 months. Multivariable logistic and ordinal regression models adjusted for demographic, aneurysmal, and treatment covariates. **Results:** TAO was used in 134 cases (54.7%; mean total duration 10.4 ± 8.7 min). In the overall cohort, TAO (presence or duration) was not independently associated with perioperative ischemia or CVS. In the SAH subgroup, cumulative TAO > 5 min conferred an approximately sixfold higher odds of ischemia (*p* = 0.012; OR 6.33), whereas no threshold was significant in incidental aneurysms. Female sex, M2 location, SAH at admission, and initial GCS < 9 independently predicted ischemia; female sex, higher ASA grade, larger size, irregular morphology, and SAH predicted CVS. SAH and aneurysm wall calcification were associated with worse 6-month mRS. **Conclusions:** TAO appears safe in elective clipping of incidental MCA aneurysms when applied judiciously, but cumulative durations beyond 5 min substantially increase ischemia risk in SAH patients. TAO management should therefore be individualized by rupture status, neurological grade, and aneurysm morphology rather than a single universal time limit.

## 1. Introduction

Middle cerebral artery (MCA) aneurysms account for approximately one fifth of all intracranial aneurysms and are frequently encountered in neurosurgical practice, reflecting both the size and branching complexity of the MCA trunk and bifurcation region. Their location at the M1 segment, bifurcation, and more distal branch points places them in close relation to eloquent cortex and critical lenticulostriate and cortical perforators, so that even limited ischemic or perforator injury can result in disabling neurological deficits. Consequently, operative strategy for MCA aneurysms must balance durable aneurysm exclusion with rigorous protection of regional blood flow [1,2,3]. Microsurgical clipping remains the preferred treatment for many MCA aneurysms, particularly those with wide necks, complex bifurcation involvement, or branch incorporation that are less amenable to endovascular techniques. Temporary arterial occlusion (TAO) of the M1 segment or parent branch is a cornerstone adjunct that softens the aneurysm sac, facilitates precise neck dissection, and mitigates the risk of uncontrolled intraoperative rupture during key steps such as proximal control, dome decompression, and clip reconstruction. However, each application of a temporary clip imposes a period of focal cerebral ischemia, and the safe duration, pattern (single vs. repeated occlusions), and site of TAO remain incompletely defined for MCA aneurysm surgery.

Clinical and neurophysiological studies suggest that brain tolerance to temporary MCA occlusion is limited, with recommendations often favoring individual occlusion times of less than 5 min, guided when available by motor-evoked potential (MEP) or somatosensory-evoked potential (SSEP) changes. Even within these nominal “safe” windows, patient-specific factors such as collateral circulation, prior ischemic burden, subarachnoid blood load, aneurysm morphology, and atherosclerotic or calcific changes at the neck can substantially alter ischemic vulnerability. Surgeons therefore frequently rely on a combination of experience-based judgment, intraoperative monitoring, and incremental clipping strategies to titrate TAO use [4,5,6].

More recently, computational approaches have been introduced to refine TAO thresholds by integrating multimodal clinical and hemodynamic variables. Shahjouei and colleagues developed an artificial neural network model to estimate safe clipping time during intracranial aneurysm surgery, reporting predicted safe intervals for MCA occlusion between approximately 90 and 950 s depending on side and flow parameters, thereby highlighting the wide interpatient variability in ischemic tolerance. Nonetheless, such models have not yet been systematically validated in homogeneous cohorts of MCA aneurysm patients undergoing contemporary microsurgical clipping with standardized TAO strategies.

Within this context, the present retrospective study focuses specifically on patients undergoing microsurgical clipping of MCA aneurysms and examines how TAO practice relates to perioperative ischemic events. The primary objective is to characterize the association between TAO use—encompassing duration, cumulative time, number of applications, and occlusion site—and radiographic or clinical ischemia in the MCA territory. Secondary aims are to identify independent patient-, aneurysm-, and surgery-related predictors of ischemia and to evaluate whether previously proposed TAO time thresholds are supported in a dedicated MCA aneurysm cohort. By emphasizing detailed operative variables and real-world microsurgical techniques, this analysis seeks to provide practical guidance for optimizing TAO use and improving outcomes in MCA aneurysm surgery.

## 2. Materials and Methods

**Figure 1:**
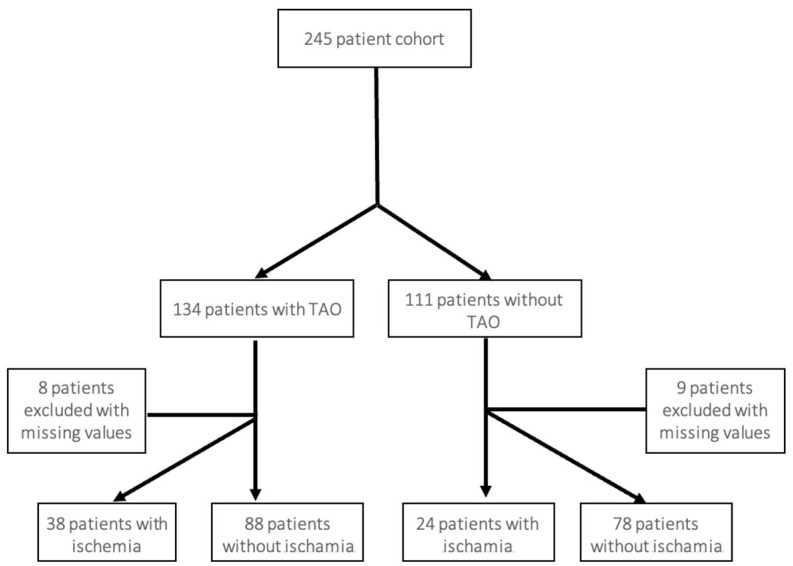
Flow chart of patient screening and analysis.

This retrospective analysis encompassed patients who underwent surgical clipping for MCA aneurysms at a tertiary neurovascular center between January 2010 and December 2020. Patients under 18 years of age, those with previous interventions on the same aneurysm, or those with incomplete medical records were excluded. A flow chart is depicted in Figure 1.

Baseline demographic data, aneurysm and hemorrhage characteristics, operative variables, and clinical outcomes were obtained from electronic medical records, operative reports, and radiology archives. Collected variables included age, sex, aneurysm size, side, and segmental location (M1, bifurcation, distal MCA), rupture status, preoperative World Federation of Neurosurgical Societies (WFNS) grade in patients with subarachnoid hemorrhage (SAH), and preoperative neurological status. Functional status was assessed using the modified Rankin Scale (mRS) at admission, discharge, and 6-month follow-up. The primary outcome was perioperative ischemia, defined as a new focal neurological deficit and/or a new hypodense lesion or diffusion restriction in the MCA territory on CT or MRI occurring within 48 h after surgery, not attributable to other causes (e.g., rebleeding or hydrocephalus). Secondary outcomes included in-hospital complications and 6-month functional outcome.

Microsurgical clipping was performed via standard pterional or frontotemporal craniotomy in all cases, with dissection of the Sylvian fissure and proximal control of the MCA according to surgeon preference and aneurysm configuration. The use of temporary arterial occlusion (TAO) of the M1 segment or relevant branch vessel was left to the discretion of the attending neurosurgeon, guided by preoperative imaging (CT angiography or digital subtraction angiography), aneurysm morphology, and intraoperative findings such as wall fragility, neck complexity, or intraoperative rupture risk. For each procedure, the presence or absence of TAO, the number of occlusion episodes, the target vessel, and the exact duration of each occlusion were recorded from the anesthetic and operative charts. For analysis, cumulative TAO duration was categorized according to prespecified thresholds: more than 5 min, more than 8 min, more than 10 min, and more than 15 min of total occlusion time.

Anesthetic management followed a standardized institutional protocol for aneurysm surgery. All patients underwent general anesthesia with endotracheal intubation, invasive arterial blood pressure monitoring, and normocapnic ventilation. In accordance with the protocol for temporary vessel occlusion, a fraction of inspired oxygen (FiO_2_) of 1.0 (100%) was administered for at least 10 min prior to the first application of a temporary clip, and mean arterial pressure was maintained at or above the patient’s baseline during TAO. Additional neuroprotective measures (e.g., mild hypocapnia, osmotherapy) were used at the anesthesiologist’s discretion in selected cases.

Screening and monitoring for cerebral vasospasm (CVS) were performed routinely in patients with aneurysmal SAH. Transcranial Doppler sonography (TCD) was used for serial assessment of flow velocities in the MCA and other basal arteries. Suspected CVS on TCD was further evaluated with digital subtraction angiography (DSA) when clinically indicated. CVS was classified as clinically relevant when elevated flow velocities consistent with vasospasm were accompanied by new neurological deficits not otherwise explained.

Statistical analyses were performed using JMP Pro 17 (SAS Institute, Cary, NC, USA). Descriptive statistics were used to summarize patient demographics, aneurysm characteristics, and outcome variables. Binary outcomes, including postoperative ischemia, clinically relevant cerebral vasospasm (CVS), and intraoperative rupture, were analyzed using logistic regression models. Ordinal logistic regression was used for ordinal clinical outcome variables, including mRS at discharge and mRS at 6 months.

For adjusted analyses, the outcome variable of interest was entered as the dependent variable, the variable under investigation was entered as the predictor of interest, and sex, smoking, anticoagulation, hypertension, wall calcification, and irregular morphology were included as covariates. Age was modeled as a continuous covariate where applicable. TAO duration was analyzed both as a continuous variable and as predefined categorical thresholds (>5, >8, >10, and >15 min).

Cases with missing data were excluded on an analysis-specific complete-case basis. As a sensitivity analysis, the primary outcome models were repeated after exclusion of cases with missing variables; effect estimates and statistical significance were materially unchanged. A two-sided *p*-value < 0.05 was considered statistically significant.

## 3. Results

### 3.1. Cohort Characteristics

The final study cohort comprised 245 patients who underwent microsurgical clipping of MCA aneurysms. Women accounted for 172 cases (70.2%) and men for 73 (29.8%), with a mean age of 55.6 ± 11.6 years. Tobacco use was reported in 126 patients (56.3%), hypertension in 136 (56.9%), and ongoing anticoagulation therapy in 64 (27.4%). Overall physiological status was moderate, with 133 patients (55.6%) classified as ASA grade 2, 50 (20.9%) as grade 3, and 28 (11.7%) as grades 1 and 4 each; ASA grade was unavailable in 6 cases. The mean aneurysm diameter was 7.6 ± 4.1 mm, with a single outlier measuring 50 mm. The M2 segment was involved in 213 aneurysms (87.3%). Aneurysm wall calcification was observed in 43 cases (17.6%), and 126 aneurysms (53.6%) demonstrated irregular morphology. Temporary arterial occlusion (TAO) was used in 134 procedures (54.7%), and intraoperative rupture occurred in 39 patients (16.1%). For regression analyses, 17 patients with missing values relevant to at least one model were excluded on an analysis-specific complete-case basis.

One hundred fifty-four aneurysms (63.0%) were incidental, whereas 91 patients (37.1%) presented with aneurysmal SAH. Among SAH patients, the most frequent Hunt and Hess grades at admission were grades 2 and 3 (23 patients [25.3%] each), followed by grade 1 in 19 (20.9%), grade 4 in 7 (7.7%), and grade 5 in 19 (20.9%). At admission, the most common mRS was 0 (145 patients [59.9%]), followed by mRS 2 in 52 (21.5%); mRS 5 was present in 23 patients (9.5%). Initial GCS was 15 in 191 patients (78.9%) and 3 in 16 (6.6%). A positive family history of intracranial aneurysm was documented in 39 patients (24.1%). Early postoperative ischemia on CT within 48 h occurred in 62 patients (25.3%), and clinically relevant CVS was diagnosed in 44 (18.6%). At discharge, 135 patients (55.8%) had an mRS of 0, which increased to 141 patients (73.1% of those with available follow-up data) at 6-month follow-up. Fifty-one patients (20.8%) were lost to follow-up at 6 months, and the overall mortality rate was 5.7% (11 patients). Patients harboring ruptured MCA aneurysms were additionally graded according to the WFNS classification at admission. All key demographic and clinical characteristics of patients with incidental (unruptured) MCA aneurysms are presented in Table 1, whereas Table 2 summarizes all corresponding details for the SAH subgroup.

### 3.2. Subarachnoid Hemorrhage Subgroup

In the SAH subgroup (n = 91), 61 patients (67.0%) were female and 30 (33.0%) were male, with a mean age of 55.4 ± 13.1 years. Current smoking was reported in 36 patients (50.7%), anticoagulation therapy in 14 (16.9%), and hypertension in 46 (53.5%). The predominant ASA grades were 3 and 4, each observed in 28 patients (32.6%). The mean aneurysm size was 8.3 ± 4.7 mm; 80 aneurysms (87.9%) were located on the M2 segment, 11 (12.1%) exhibited wall calcification, and 37 (45.1%) had irregular morphology. TAO was employed in 62 cases (68.1%), and intraoperative rupture occurred in 25 patients (28.1%). New post-treatment neurological deficits were documented in 36 patients (46.8%).

At 6-month follow-up, the most frequent mRS was 0 (26 patients [44.8%]), followed by mRS 1 in 10 (17.2%). A positive family history of aneurysm was present in 4 patients (7.8%). Postoperative ischemia occurred in 33 patients (41.2%), and CVS was diagnosed in 29 (33.0%). Demographic and clinical characteristics of the SAH cohort at admission are provided in Table 2.

**Table 2:** Baseline demographics of the ruptured MCA aneurysm cases (N = 91).

SAH Demographics	N (%)
Total number of patients	91
Gender Distribution	
- Female	61 (67.0%)
- Male	30 (33.0%)
Mean Age (years)	55.4 ± 13.1
Smoking	36 (50.7%)
Anticoagulation Therapy	14 (16.9%)
Hypertension	46 (53.5%)
ASA Grade	
- Grade 3	28 (32.6%)
- Grade 4	28 (32.6%)
Mean WFNS Score	2.6 ± 1.7
WFNS I°	38 (41.8%)
WFNS II°	15 (16.5%)
WFNS III°	2 (2.2%)
WFNS IV°	12 (13.2%)
WFNS V°	22 (24.2%)
Mean Aneurysm Size (mm)	8.3 ± 4.7
Aneurysm Location	
- M2 Segment	80 (87.9%)
Aneurysm Wall Calcification	11 (12.1%)
Irregular Aneurysm Morphology	37 (45.1%)
TAO for Rupture Prophylaxis	62 (68.1%)
Intraoperative Rupture	25 (28.1%)
Post-treatment Deficits	36 (46.8%)
Mean mRS at discharge	2.3 ± 2
mRS at 6 months	
- mRS 0	26 (44.8%)
- mRS 1	10 (17.2%)
- mRS 2	5 (8.6%)
Positive Family History	4 (7.8%)
Ischemia	33 (41.2%)
Clinically Relevant CVS	29 (31.9%)
Mortality	10 (11%)

### 3.3. Predictors of Ischemia, Cerebral Vasospasm, and Outcome

On multivariable analysis, female sex (*p* = 0.0195), aneurysm location on the M2 segment (*p* = 0.0136), presence of SAH at admission (*p* < 0.0001), and an initial GCS less than 9 (*p* < 0.0001) were independently associated with an increased risk of perioperative ischemia. TAO use per se was not significantly associated with ischemia in the overall cohort (*p* = 0.4605), although a time-dependent effect was identified within the SAH subgroup (see below).

Risk factors for clinically relevant CVS included female sex (*p* = 0.0474), ASA grades 2 and 4 (*p* = 0.0202 and *p* = 0.0413, respectively), larger aneurysm size (*p* = 0.0413), irregular aneurysm morphology (*p* = 0.0078), presence of SAH (*p* < 0.0001), and initial GCS less than 9 (*p* < 0.0001). TAO was not significantly associated with CVS (*p* = 0.4152). For ASA grade, dummy variables were entered into the regression model with ASA 1 as the reference category; the reported *p* values correspond to the regression coefficients for grades 2 and 4.

Anticoagulation therapy at admission (*p* = 0.0298), presence of SAH (*p* < 0.0001), admission mRS 2 and 3 (*p* = 0.0204), and initial GCS less than 15 (*p* = 0.0363) were associated with poor clinical outcome at discharge. At 6-month follow-up, independent predictors of worse functional outcome were aneurysm wall calcification (*p* < 0.0001) and presence of SAH (*p* < 0.0001).

### 3.4. TAO Duration Thresholds

TAO was performed in 134 patients (54.7% of the total cohort); their demographic and clinical characteristics are summarized in Table 3. Cumulative TAO duration exceeded 5 min in 84 patients (62.7%), 8 min in 53 (44.5%), 10 min in 40 (34.2%), and 15 min in 24 (20.5%). The mean total TAO time was 10.4 ± 8.72 min, with a median of 8 min.

Patients were stratified into four groups according to cumulative TAO duration (>5, >8, >10, and >15 min) and further subdivided by rupture status (incidental vs. SAH). In the SAH subgroup, exceeding a cumulative TAO duration of 5 min was significantly associated with higher odds of postoperative ischemia, with an approximately sixfold increase in risk (*p* = 0.012; OR 6.33). No TAO duration threshold showed a significant association with ischemia in the incidental aneurysm cohort. These associations remained robust when TAO duration thresholds were entered into multivariable models together with established risk factors (including sex, age, aneurysm location, and admission GCS), indicating that prolonged TAO represents an independent predictor of ischemia, specifically in patients with SAH.

Within the SAH subgroup, age and sex were additionally associated with postoperative ischemia (*p* < 0.05). In contrast, TAO duration did not correlate with the occurrence of clinically relevant CVS in either incidental or SAH patients, irrespective of the thresholds applied.

### 3.5. TAO and Intraoperative Rupture

In univariate analysis, intraoperative aneurysm rupture was significantly associated with an increased risk of postoperative ischemia (*p* < 0.001; OR 2.5 [95% CI 2.28–2.76]). This association persisted after adjustment for sex and age (*p* < 0.05; OR 2.1 [95% CI 1.009–4.41]).

Use of TAO was associated with intraoperative rupture in adjusted exploratory models (*p* < 0.05; OR 4.4 [95% CI 1.19–16.3]). However, because operative documentation did not reliably establish whether TAO was applied before or after rupture, this association cannot be interpreted as causal. A similar pattern was observed in an exploratory model additionally adjusted for SAH status, aneurysm size, aneurysm wall calcification, and irregular morphology, suggesting that TAO use may be more frequent in technically demanding or unstable aneurysms rather than being an independent cause of rupture.

## 4. Discussion

TAO has long been a key adjunct in microsurgical management of MCA aneurysms, yet its safety profile and optimal duration remain subjects of ongoing debate. In this cohort, the use of TAO was not associated with a significant increase in postoperative ischemia in the overall population, supporting its relative safety in elective clipping of incidental MCA aneurysms when used judiciously. This finding challenges the traditional concern that even moderately prolonged temporary clipping is inherently hazardous and instead supports the concept that TAO can be safely employed as needed, provided that meticulous surgical technique and brain protection strategies are applied.

The absence of a significant ischemia cutoff across the predefined TAO duration thresholds (5, 8, 10, and 15 min) in the total cohort is noteworthy. With a median cumulative TAO time of 8 min and a mean of 10.4 min, extended temporary occlusions did not translate into a statistically higher ischemia rate in electively treated aneurysms. This observation questions the dogmatic adherence to a strict 5 min limit and is consistent with prior work suggesting that tolerable occlusion times may range from a few minutes up to 10–15 min, particularly in patients with robust collateral circulation and optimized intraoperative management [7]. At the same time, the guiding principle in practice should remain that temporary clipping is maintained “as short as possible, but as long as necessary,” with careful real-time assessment of surgical progress and perfusion-related risk [8,9,10].

In contrast, the statistically significant 5 min threshold identified in the SAH subgroup indicates a substantially narrower safety margin in the acutely injured brain. The approximately sixfold increase in ischemia risk beyond 5 min of cumulative TAO in SAH patients suggests that subarachnoid blood burden, impaired autoregulation, and early microvascular dysfunction render these patients more vulnerable to even brief episodes of focal hypoperfusion [11,12]. This underscores the need for particularly conservative TAO strategies in ruptured aneurysms, including limiting individual occlusion periods, frequent clip release, optimization of oxygen delivery and blood pressure, and, where available, integration of advanced intraoperative monitoring such as motor- and somatosensory-evoked potentials or quantitative perfusion monitoring [13]. Future prospective studies focusing on SAH patients, ideally with standardized neuromonitoring protocols, are warranted to refine time- and context-dependent TAO thresholds in this high-risk subgroup [7,14,15].

Importantly, TAO was not associated with clinically relevant CVS in either the overall cohort or subgroup analyses. This suggests that transient, intraoperative proximal occlusion is unlikely to be a major driver of delayed vasospasm, which is more plausibly related to subarachnoid blood load, inflammatory cascades, and patient-specific vascular susceptibility. The lack of correlation between TAO and CVS further supports the interpretation that TAO-related ischemic risk is predominantly an early, focal, and hemodynamic phenomenon rather than a trigger for delayed vasculopathy.

Beyond TAO-specific effects, this study identified several independent risk factors for perioperative ischemia and CVS. Female sex emerged as a consistent risk factor for both complications, implying potential sex-related differences in vascular biology, hormonal milieu, or collateral capacity that merit further investigation. Aneurysm location on the M2 segment was associated with increased ischemia risk, likely reflecting the greater density of eloquent cortical and perforating branches at these bifurcation and branch points and the narrower margin for clip-related or ischemic compromise. The presence of SAH at admission was the strongest predictor of both ischemia and CVS, reinforcing the established concept that aneurysmal SAH initiates a cascade of inflammatory and vasoreactive changes that heighten vulnerability to both early infarction and delayed cerebral ischemia. Likewise, an initial GCS below 9 was strongly associated with ischemia and CVS, indicating that patients with poor neurological grade at presentation require particularly intensive intraoperative and postoperative management.

Functional outcome was driven predominantly by the severity of initial hemorrhage and aneurysm pathology. SAH at admission remained a powerful predictor of poor outcome at discharge and at 6-month follow-up, underscoring the lasting impact of initial brain injury and systemic derangement on long-term recovery. In addition, aneurysm wall calcification was independently associated with worse 6-month outcomes. Calcification likely reflects advanced atherosclerotic change and may increase technical complexity, limit clip repositioning options, and predispose to distal embolization or parent vessel compromise. These findings suggest that detailed assessment of aneurysm wall characteristics on preoperative imaging should inform risk stratification and operative planning, and that calcified aneurysms may particularly benefit from heightened intraoperative vigilance and postoperative surveillance for ischemic sequelae [16].

The observed association between TAO use and intraoperative rupture in adjusted exploratory models should be interpreted with caution. Given that operative documentation did not reliably establish whether TAO preceded or followed rupture, and that surgeons are more likely to deploy temporary clips in anatomically complex, fragile, or hemodynamically unstable aneurysms, TAO likely functions as a surrogate marker of case complexity rather than a direct cause of rupture. Similarly, intraoperative rupture itself was strongly associated with postoperative ischemia, consistent with the notion that rupture reflects a high-risk operative course with potential for abrupt hypotension, loss of visualization, perforator injury, and more aggressive clip application. Together, these findings emphasize that the relationship among TAO, rupture, and ischemia is confounded by underlying aneurysm complexity and intraoperative difficulty; careful case selection, early proximal control, and structured rupture-management protocols remain central to mitigating these risks.

### Limitations

This study has several important limitations. Its retrospective, single-center design introduces potential selection bias and limits generalizability, particularly with respect to institutional surgical philosophy and anesthetic protocols. Although rigorous, standardized data extraction and multivariable modeling were employed, residual confounding by unmeasured factors such as detailed perforator anatomy, surgeon experience, intraoperative blood pressure variability, or unrecorded neuromonitoring changes cannot be excluded. Assessment of TAO exposure depended on operative and anesthetic documentation, which may incompletely capture intermittent versus cumulative occlusion; use of the longest or cumulative recorded duration likely biases any misclassification toward underestimating true effects. Furthermore, the temporal relationship between TAO and intraoperative rupture could not be reconstructed with sufficient precision to establish causality, necessitating interpretation of TAO primarily as an indicator of operative complexity. Finally, early ischemia was defined within a 48 h imaging window and might reflect mixed mechanisms, including perforator compromise, thromboembolism, clip-related stenosis, or SAH-related microvascular injury, rather than TAO alone. Despite these constraints, the focus on a homogeneous MCA aneurysm cohort, systematic categorization of TAO durations, and parallel analysis of elective and SAH cases provide clinically relevant insights that can inform operative strategy and guide the design of future prospective, multimodal monitoring studies.

## 5. Conclusions

In this large, homogeneous cohort of patients undergoing microsurgical clipping of MCA aneurysms, temporary arterial occlusion was not associated with an increased risk of perioperative ischemia in the overall population, supporting its judicious use as a safe adjunct in elective surgery for incidental aneurysms when combined with standardized brain protection strategies. At the same time, the identification of a statistically significant 5 min threshold for cumulative TAO duration in patients with aneurysmal SAH indicates a substantially narrower ischemic tolerance in the acutely injured brain and argues for particularly conservative occlusion strategies in this high-risk subgroup. Taken together, these findings refine existing empirical time limits for temporary clipping, suggesting that individualized TAO management should be guided not only by duration but also by rupture status, neurological grade, and collateral reserve rather than by a single universal cutoff.

Beyond TAO itself, this study delineates a set of clinically relevant predictors of ischemia, vasospasm, and outcomes—including female sex, M2 location, poor initial GCS, aneurysmal SAH, and aneurysm wall calcification—that can be readily incorporated into preoperative risk stratification and intraoperative decision-making. The observation that calcified aneurysms are independently associated with worse long-term outcomes further underscores the importance of detailed morphological assessment in planning clip application and anticipating postoperative surveillance needs. By providing granular, MCA-specific data on TAO practice and perioperative risk, these results offer pragmatic guidance for optimizing temporary clipping in contemporary microsurgical aneurysm surgery and highlight the need for prospective, multimodal monitoring studies to validate tailored TAO thresholds, particularly in patients with ruptured MCA aneurysms.

## Figures and Tables

**Table 1:** Baseline demographics of the unruptured MCA aneurysm cases.

Demographics	N (%)
Total number of patients	154
Gender distribution	
Male	43 (27.9%)
Female	111 (72.1%)
Mean Age	55.7 ± 10.6
Smoking	91 (59%)
Hypertension	90 (58.4%)
Anticoagulation	50 (32.5%)
Mean aneurysm size in mm	7.72 ± (5.57)
Aneurysm location	
M2-segment	134 (87%)
Aneurysm Wall Calcification	32 (20.8%)
Irregular Morphology	89 (57.8%)
TAO performed	72 (46.8%)
Ischemia on CT within 48 h	29 (18.8%)
Cerebral Vasospasm	15 (9.7%)
Mean mRS at discharge	0.34 ± 0.9
mRS 0	119 (77.3%)
mRS 1	22 (14.3)
mRS 2	5 (3.2%)
Clinically relevant CVS	15 (9.7%)
Mortality	1 (0.6%)

**Table 3:** Demographic overview of cases, where TAO was performed.

TAO Performed	N (%)
Total number of patients	134
Gender distribution	
Female	88 (65.7%)
Mean age	56 ± 12
Smoking	66 (49.3%)
Anticoagulation	35 (26.1%)
Hypertension	74 (55.2%)
Irregular morphology	66 (49.3%)
Mean Hunt and Hess grade	1.37 ± 1.8
SAH upon admission	62 (46.3%)
mRS at discharge	1.4 ± 1.84
mRS at 6 months	0.97 ± 1.85

## Data Availability

The data presented in this study are available on request from the corresponding author due to privacy and ethical restrictions.
